# To thine own test be true: HIV self‐testing and the global reach for the undiagnosed

**DOI:** 10.1002/jia2.25256

**Published:** 2019-03-25

**Authors:** Vincent Wong, Erin Jenkins, Nathan Ford, Heather Ingold

**Affiliations:** ^1^ United States Agency for International Development (USAID) Global Health Bureau Office of HIV/AIDS Prevention Care and Treatment Division; ^2^ HIV/AIDS Department Organisation mondiale de la Sante Geneva Switzerland; ^3^ Unitaid Geneva Switzerland

**Keywords:** HIV self‐testing, HIV policy, VCT, PITC, HIV diagnosis

Globally, we are at an inflection point in achieving UNAIDS’ 95‐95‐95 goals for 2030. A recent *Lancet* editorial observed that “the last big shared challenge remaining is testing—in every region the number of undiagnosed HIV infections remains a substantial barrier to achieving UNAIDS targets and ending AIDS by 2030” 1. While UNAIDS estimates we are at 75% diagnostic coverage globally, within this figure is great variation: between men and women, younger and older individuals, rural and urban populations, among key populations and between countries 2, 3. After 18 years of expansive programming in global health for HIV testing through a multitude of modalities in communities and facilities, reaching the remaining undiagnosed individuals with flat‐lined donor funding will require new efforts 3. Many of the remaining undiagnosed individuals are presumably not engaging with HIV services, and novel avenues to HIV testing services (HTS) that overcome both stigma and structural barriers are needed: a new HIV testing paradigm is urgently needed to reach these remaining undiagnosed individuals and effectively link them to treatment.

HIV self‐testing (HIVST) has developed substantially in recent years and is now considered a new and critical HIV response strategy in controlling the epidemic. In 2012, the US FDA approved the OraQuick^®^ HIV Self‐Test Kit introducing the first HIV rapid test kit intended for use by the general population and available for purchase over‐the‐counter in the United States. Building on a history of public health interventions aimed at self‐screening for health conditions that includes home pregnancy tests, breast self‐examinations for cancer screening and blood‐glucose monitoring, access to HIVST permits individuals perceiving themselves to be at‐risk of infection to test independently and privately. Global attention to the potential of HIVST took root in 2013 following the OraQuick FDA approval, with UNAIDS and the World Health Organization (WHO) holding an initial consultation on the ethical and public health implications of HIVST. At that time, no HIVST kits were publicly available in low‐ and middle‐income countries (LMIC) (outside a small number of studies and “grey market” test kits), no normative guidelines had been established and only a small body of LMIC‐focused evidence around HIVST existed. Within a year, a full HIVST journal supplement had been published exploring early issues in HIVST introduction: regulation and policy, optimal product profiles, ethical considerations and both positive and negative potential impacts of rollout 4, 5, 6. This led, in 2015, to Unitaid's catalytic investment in the five‐year HIV Self‐Testing Africa (STAR) initiative. The first two‐year phase, which included Malawi, Zambia and Zimbabwe, aimed to generate evidence on the feasibility and acceptability of HIVST as well as how to distribute self‐test products effectively, ethically and efficiently, with adequate post‐test support. Evidence from this phase supported country policy development, and studied impacts and cost‐effectiveness of various delivery models, addressed structural barriers and assessed consumer demand. Findings from these and other studies led to the WHO guidance on HIVST in 2016 (also strategically paired with the HTS) Partner Notification guidelines 7. A separate but concurrent process resulted OraQuick being the first WHO prequalified HIVST kit. In 2017, the Bill and Melinda Gates Foundation provided financial support to bring the unit cost of the OraQuick self‐test kit down to US$2 in selected sub‐Saharan African and other low‐income countries, removing a critical cost barrier to HIVST expansion 8.

These initial supportive efforts established the foundation needed for HIVST expansion across countries. The second three‐year phase of STAR added Lesotho, Eswatini and South Africa, and aimed to create a market for HIVST and evaluate optimal distribution models for increasing access to testing among those unwilling or unable to utilize traditional testing venues and ensuring linkage from a preliminary positive HIVST result to confirmatory testing and treatment. By the end of the programme, Unitaid, the STAR programme's commodities funder, will have provided five million HIVST kits to the six project countries. Building on STAR's momentum and their own smaller scale pilot programmes in 2016, PEPFAR expanded its HIVST programming and will have delivered 2.3 million HIVST kits across 11 countries in 2017 to 2018. Similarly, the Global Fund is expanding HIVST support across 18 countries, estimated to cover about 12% of the global HIVST volume 9. With 59 countries having, or developing, national HIVST policies, there is global acceleration towards the expansion of HIVST access and programmes and, with good linkage, increased diagnostic coverage 9. However, these numbers remain small relative to the overall number of people tested; PEPFAR alone accounted for roughly 85 million HIV tests in 2017 10. But HIVST deployed strategically within programmes, and made available through multiple avenues, is anticipated to amplify the impact of current HIV programming by reaching the critical remaining at‐risk populations with needed testing and treatment.

The articles collected for this *Supplement* present a diverse range of the key findings from the first phase of STAR, and provide a basis for needed programmatic action to accelerate expansion. Presently, in sub‐Saharan Africa, there are 15 countries that have HIVST policies in place or under consideration and multiple products available with some type of certification 9, 11. However, products of unknown quality have been available on the unregulated market, posing risks and underscoring the need for further quality and consumer protection regulations 6, 11. Dacombe *et al*. explore the regulatory environment in Malawi, Zambia and Zimbabwe 12. Using key informant interviews which included laboratory staff and policymakers, they consulted 66 individuals from the three countries. Interviews showed that in these countries, there was a need for regulation of *in vitro* diagnostic tests in general, and HIVST kits were no exception. The authors call for a regional collaboration to spread the regulatory burden across countries and facilitate the passing of required legislation to support more codified regulation of diagnostics.

WHO prequalified test kits have gone through quality assurance evaluations aimed at ensuring “global standards of quality, safety and efficacy” to support Ministries of Health and the introduction of quality diagnostics 13. However, product performance includes not just quality standards of the test kit itself, but also usability by the target population and the successful insertion of HIVST into the clinical cascade. Early studies showed some challenges in following instructions for use (IFU) 5, 14, but as kits have been refined, results have improved. A recent review demonstrated general agreement between results of HIVST kits and facility testing algorithms 15. However, challenges relating to literacy remain, underscoring the need for clear and simple language in package inserts 15, 16, 17 and IFUs that are adapted to local contexts. In this *Supplement*, Simwinga *et al*. present findings from Malawi and Zambia evaluating an IFU translated into the local language and evaluated for clarity and ease of use 18. Investigators used feedback from testers to optimize the IFU, concurring with previous findings that the educational level of the tester correlates to the ability to follow the IFU. In response, they suggest that in certain contexts community demonstrations of how to use HIVST kits could overcome this barrier.

In another study, given that programmes have proposed late reading of returned kits to determine HIV positivity, Watson *et al*. evaluated the OraQuick HIV‐1/2 antibody test kits for result stability post‐testing. They showed that while strongly reactive HIVST remained stable, 29% of initially non‐reactive kits converting to be weakly reactive false positive when read at least four days later, countering previous work which indicated OraQuick test kits were stable for up to a year 19, 20. Re‐reading may be problematic and result in artificially inflated positivity rates; this finding led the WHO to recommend *against* any delayed readings of kits 21.

Eaton *et al*. model how HIVST and other “test for triage” strategies might impact national algorithm performance 22. Considering modelled high‐ and low‐prevalence scenarios, as well as using data from Malawi, a high‐prevalence country with high rates of diagnosis 23, the authors show that the addition of triage testing before the national algorithm increases the positive predictive value and decreases the number of false‐positive diagnoses, possibly eliminating the need for verification testing at initiation of antiretroviral therapy (ART).

Methodologies for HIVST distribution will be a critical aspect of programme effectiveness. Various delivery modes have been considered: vending machines, over‐the‐counter at pharmacies, secondary distribution when an HIVST is distributed to one person for use by another, and facility‐ and community‐based distribution 11, 15, 24, 25, 26. In this *Supplement*, Sibanda *et al*. began with the clients, investigating preferences for access to HIVST in rural Zimbabwe through discrete choice assessments, finding respondent preferences for door‐to‐door distribution, kits free‐of‐charge, access by telephone to help in using kits and linkage to confirmatory testing, and that programmes use patient reminders and outreach to enhance effectiveness 27. For confirmatory testing and ART initiation, respondents also preferred these to be free, located near their home and that ART could be initiated immediately 27. This study supports previous findings on user preferences emphasizing ease of access, usability and privacy 11.

Also in this *Supplement*, Hatzold *et al*. reviewed STAR data from Malawi, Zambia and Zimbabwe that assessed the integration of HIVST tools into HIV programming 28. They found that by having clients perform HIVST in outpatient settings, they were able to decongest clinical testing facilities because healthcare workers could focus only on those that screened positive. They also demonstrated the ability to reach men through community distribution and in particular workplaces, and explored male attitudes to HIVST, noting that the briefer counselling messages, privacy and convenience appealed to them 28.

Advantages of HIVST, such as the ability to test privately, may also be misused or abused and potential social harms should not be ignored. Previously in Kenya, low rates of physical and verbal abuse have been reported with the introduction of HIVST kits by women for their male partners to test themselves 29. In this *Supplement*, Kumwenda *et al*. provide new evidence on social harms from projects in Malawi, summarizing data from six HIVST projects from 2011 to 2017 where a combination of qualitative and quantitative methods were used 30. Coercion was reframed to have both negative and positive aspects, and the concept of compassionate coercion was introduced to describe instances when family members encourage a member who is ill to test. Overall, they report 25 serious adverse events through the active reporting systems from all six studies with a total of 178,833 self‐tests distributed. The most common event was marriage breakdown in serodiscordant relationships though verbal abuse, and physical and economic intimate partner violence were infrequently also observed 30. The potential for social harms is not unique to HIVST, but the present work elucidates the need for intimate partner violence screening when considering HIVST secondary distribution and partner testing, and the need for ongoing monitoring of social harms within existing systems.

In the context of HIVST, linkage to care refers not just to the initiation of ART, but first to confirmatory testing after a positive HIVST 31. Since using an HIVST kit in private is often preferred by testers, the onus to link to care is firmly placed in the hands of the tester. As such, linking testers to care and estimating linkage rates can be a challenge. Some HIVST research studies have estimated linkage rates to be between 36% and 78% with a variety of methodologies as there is no standard process to measure linkage 24, 29, 31, 32. In this *Supplement*, Neuman *et al*. reflect on the difficulties in estimating linkage as HIVST is brought to scale 33. They note the limited metrics available – HIVST kit distribution totals and self‐reported data, neither of which is optimal to estimate linkage rates accurately 33. The investigators present a summary of study protocols estimating linkage from published STAR studies. These estimate HIVST linkage by using ecological indicators such as comparisons of ART initiation rates in areas with HIVST campaigns versus in areas without HIVST campaigns. Taken on its own, it is only correlative; however, when considered in addition to other information it can be used to create a body of evidence regarding linkage to care.

Costing HTS is highly contextual with considerable variation, but important to programme planning and bringing HIVST to scale. In this *Supplement*, Mangenah *et al*. performed a cost analysis of community HIVST kit distribution in Malawi, Zambia and Zimbabwe as well as a sensitivity and scenario analysis to project future costs 34. The average cost per kit distributed (i.e. not only the commodity cost) ranged from US$ 7.23 to US$ 14.58 with variation by site location, but still comparable to previously published values 35. In a second article, Cambiano *et al*. use this data to present a modelling analysis comparing community distribution to three priority populations in Zimbabwe and Malawi: women having transactional sex (WTS), youth and adult men 36. The model showed that distribution to men averted the most deaths, but distribution to WTS was the most efficient as measured in number of tests per death averted. Cambiano *et al*. have added to cost‐effectiveness research, considering the trade‐offs between investing in HIVST and other HIV programmes, they estimate that HIVST is cost‐effective when the mean cost per disability adjusted life year averted is below US$ 500. According to their models, this occurs when HIVST kits were distributed to WTS and men but not to youth.

Offering commentary on the use of HIVST, Ingold *et al*. focus on the broader policy environment in LMIC, market development for HIVST kits, the STAR programme experience and its positioning for HIVST scale‐up 37. As part of the intervention, the STAR programme engaged with manufactures and stakeholders at the country and multilateral levels to create demand, assess viability of HIVST as a route to diagnosis and research delivery methods. The overall goal being to pave the way for increased access to quality HIVST kits to mobilize more people living with HIV to know their status. The authors highlight the progress that has been made in addressing these barriers, including amassing a sufficient evidence base for WHO guidance and a more enabling policy environment in general, prequalification of two types of HIVST kits and a more robust product pipeline. Pilot studies have demonstrated the ability of HIVST to reach populations that have traditionally been refractory to other testing strategies and viability for priority populations. Ingold *et al*. also touch on outstanding challenges to be addressed and present a call to action to maintain momentum in bringing HIVST to scale 37.

Since 2012, substantial progress has been made on HIVST programmes, policy and products – but few countries are implementing HIVST at scale, with many still conducting smaller volume pilot programmes. Recent HIV response framings have declared that “what got us here won't get us there” 38; for HIV testing, the rapid expansion of voluntary counselling and testing, provider‐initiated approaches and community‐based campaigns have achieved a global 75% diagnosis rate. The final reach to the remaining undiagnosed individuals, including early diagnosis of decreasing numbers of newly infected persons, will depend critically on an evolution of new approaches: HIVST, expansions of index testing and partner notification as a new minimum standard of care, and programmatic improvements of existing HTS access points. The evidence to date has demonstrated the potential of HIVST to reach both the unreached and those at high risk, which is key in achieving the 95‐95‐95 goals and controlling the epidemic.

However, operational questions remain. Intentional misuse, accuracy and performance of secondary distribution, more effective leveraging of public–private sector collaborations to reach high‐risk populations, programmatic use of blood‐based HIVST kits, use of HIVST as a demand generation tool for Pre‐exposure prophylaxis (PrEP), volume procurement approaches to reduce unit pricing and the use of mobile technology and other methods to estimate linkage post‐HIVST, could all benefit from more operations research to guide programming.

The body of evidence produced in this *Supplement* adds significantly to the field of HTS, exemplifying the potential public health role of this new technology to critically increase coverage. Still, HIVST access will not reach the scale needed to impact the epidemic without both leveraging existing health programmes and developing new and innovative avenues of access. The integration of HIVST should work to amplify existing HIV programming to achieve multiple purposes that serve public health goals: reaching unreached and high‐risk individuals at an early disease stage, reducing testing burdens on taxed health systems, and critically identifying the most effective avenues to linking persons screening positive to onward testing and treating or linking persons screening negative to prevention services. All are outcomes of critical importance to controlling the HIV epidemic. And novel avenues such as private sector delivery will continue to need to be explored. While we find ourselves at the “last big shared challenge” of HIV testing, in the race towards control of the HIV epidemic, this *Supplement* strongly illustrates that a new testing paradigm based in part on HIVST is key to the next decade of the HIV response and achieving 95% diagnosis rates everywhere.

## Competing interests

VW, EJ, NF and HI declare no competing interests.

## Authors’ contributions

VW and EJ drafted the initial manuscript. All authors critically reviewed the manuscript, suggested revisions and editorial changes, and approved the final version.

VW is a member of the Unitaid HIVSTAR Technical Advisory Group.
